# Fascia Only Anterolateral Thigh Flap for Coverage of a Dorsal Foot Defect After Sarcoma Excision

**Published:** 2017-03-11

**Authors:** Matthew Miller, Mitchell A. Pet, William P. Schmitt, Shannon M. Colohan

**Affiliations:** University of Washington Medical Center, Seattle, WA

**Keywords:** anterolateral thigh flap, fascia flap, sarcoma, dorsal foot, free tissue transfer

## DESCRIPTION

A 48-year-old woman presented with a 2-month history of a painless mass growing on her foot. Excisional biopsy showed pleomorphic soft tissue sarcoma with positive deep and peripheral margins. The patient underwent adjuvant radiation therapy followed by wide local excision and reconstruction with an ipsilateral fascia-only anterolateral thigh flap and split-thickness skin graft.

## QUESTIONS

1. **What is the typical clinical presentation for extremity soft tissue sarcoma?**
2. **What are the preferred treatment modalities for extremity soft tissue sarcomas?**
3. **What is the blood supply to the anterolateral thigh flap?**
4. **How can anterolateral thigh flap harvest be tailored when a traditional fasciocutaneous flap is too thick or too thin for the defect?**

## DISCUSSION

The exact etiology of soft tissue sarcoma is uncertain, but most arise de novo rather than from a preexisting lesion. However, certain risk factors have been described. A genetic predisposition to these sarcomas is found in Li-Fraumeni syndrome as well as neurofibromatosis type I. Prior radiation or chemotherapy increases the likelihood of developing soft tissue sarcomas. Chronic inflammation and long-standing lymphedema have been associated with certain types of sarcomas, as seen in Stewart-Treves syndrome. Most patients with soft tissue sarcoma initially present with a painless mass gradually increasing in size. Excisional or incisional biopsy is chosen on the basis of size and location. If suspicion for sarcoma is moderate or high, biopsy should be performed by a surgical oncologist. In any case, the incision for biopsy should be planned such that it can be completely excised during potential subsequent tumor extirpation.^1^

The preferred method of treating most extremity soft tissue sarcomas includes combined surgery and radiation. Radiation usually precedes definitive surgery, and as such, vascularized soft tissue coverage is commonly indicated. The type of tissue surrounding the sarcoma determines the necessary margins; 1- to 2-mm margins are suitable for fascia whereas fat and muscle may require up to 1-cm margins.^2^

The fasciocutaneous anterolateral thigh flap is an excellent choice for reconstruction of many sarcoma defects because it has a long pedicle, minimally morbid donor site, and often lends itself to concurrent harvest. This flap is based on musculocutaneous and/or septocutaneous perforators from the descending branch of the lateral circumflex femoral artery, which itself is a branch of the profunda femoris.^3^

Modifications of this flap allow considerable tailoring to the defect. For defects with extensive dead space, a portion of the vastus lateralis can be included for bulk.^4^ This compound flap is particularly easy to harvest, as no intramuscular dissection is necessary. For defects requiring a thin flap, the fasciocutaneous anterolateral thigh flap can be thinned primarily.^5^ It can also be elevated at the level of the superficial fascia or taken as a fascia only flap.^5^^,^^6^

In this case, the primary reconstructive challenge was to provide durable coverage of exposed bone and tendon within a radiated field, while avoiding excessive dorsal foot bulk, which would preclude the use of normal footwear. As the resected specimen was only a few millimeters thick, we chose the thinnest of the options above, a fascia only flap covered by split-thickness skin graft. Technical pearls for harvest of this flap include avoiding cauterization and maintaining a thin stippling of fat on top of the fascia to improve skin graft take, harvesting extra fascia in anticipation of some primary contraction, and identification of perforator vessels in the suprafascial plane as a means of identifying underlying perforators to the flap. A meshed split-thickness skin graft was chosen to minimize the metabolic demand of the graft on top of the fascia, which is not as densely vascularized as some other wound beds. Postoperative care included 5 days of bed rest, followed by a slowly advancing regimen of dependent positioning. Weight bearing with physical therapy was commenced at 3 weeks postoperatively.

## Figures and Tables

**Figure 1:**
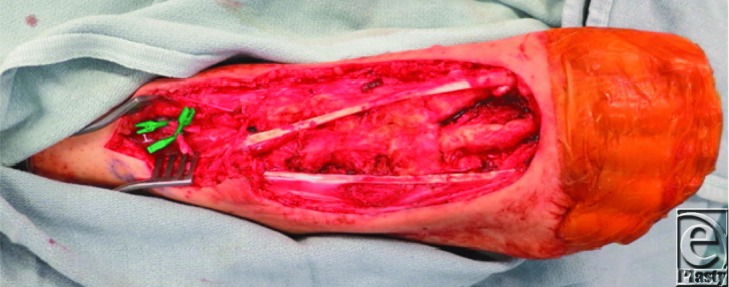
Dorsum of right foot following sarcoma excision. The extensor tendons of the second and third toes were involved and included with the specimen. The extensor hallucis longus and fourth toe extensor tendons, in addition to metatarsal bone, are exposed in the wound. The anterior tibial vessels have been prepared for microvascular anastomosis.

**Figure 2:**
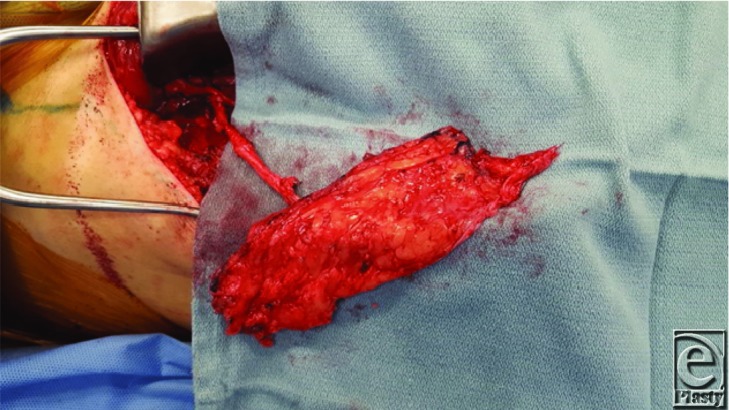
Fascia only anterolateral thigh flap before division of the pedicle.

**Figure 3:**
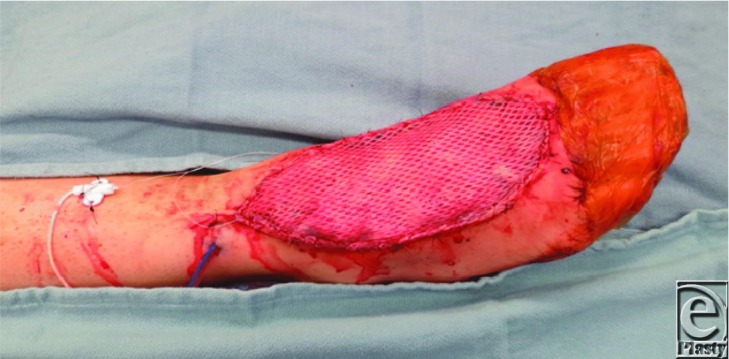
Anterolateral thigh flap after microvascular anastomosis, inset, and coverage with a meshed split thickness skin graft.

**Figure 4:**
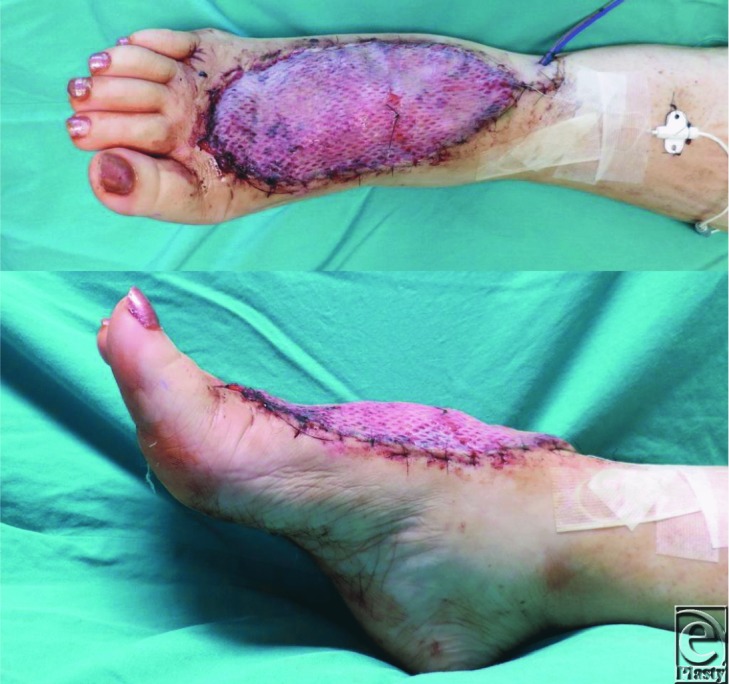
Appearance of the flap with 100% take of the skin graft, at first dressing change on postoperative day 5.
